# USP1 deubiquitinase: cellular functions, regulatory mechanisms and emerging potential as target in cancer therapy

**DOI:** 10.1186/1476-4598-12-91

**Published:** 2013-08-10

**Authors:** Iraia García-Santisteban, Godefridus J Peters, Elisa Giovannetti, Jose Antonio Rodríguez

**Affiliations:** 1 Department of Genetics, Physical Anthropology and Animal Physiology, University of the Basque Country UPV/EHU, Leioa, Spain; 2 Department of Medical Oncology, VU University Medical Center, Amsterdam, The Netherlands

**Keywords:** Deubiquitinase, USP1, DNA damage, Chemoresistance

## Abstract

Reversible protein ubiquitination is emerging as a key process for maintaining cell homeostasis, and the enzymes that participate in this process, in particular E3 ubiquitin ligases and deubiquitinases (DUBs), are increasingly being regarded as candidates for drug discovery. Human DUBs are a group of approximately 100 proteins, whose cellular functions and regulatory mechanisms remain, with some exceptions, poorly characterized. One of the best-characterized human DUBs is ubiquitin-specific protease 1 (USP1), which plays an important role in the cellular response to DNA damage. USP1 levels, localization and activity are modulated through several mechanisms, including protein-protein interactions, autocleavage/degradation and phosphorylation, ensuring that USP1 function is carried out in a properly regulated spatio-temporal manner. Importantly, USP1 expression is deregulated in certain types of human cancer, suggesting that USP1 could represent a valid target in cancer therapy. This view has gained recent support with the finding that USP1 inhibition may contribute to revert cisplatin resistance in an *in vitro* model of non-small cell lung cancer (NSCLC). Here, we describe the current knowledge on the cellular functions and regulatory mechanisms of USP1. We also summarize USP1 alterations found in cancer, combining data from the literature and public databases with our own data. Finally, we discuss the emerging potential of USP1 as a target, integrating published data with our novel findings on the effects of the USP1 inhibitor pimozide in combination with cisplatin in NSCLC cells.

## Introduction

Ubiquitin is a 76 amino acid long peptide that can be covalently attached to proteins to modulate their stability, localization or function. Initially identified as a “destruction tag” leading to the degradation of the modified protein in the proteasome [1], it became later evident that ubiquitin conjugation also causes a variety of non-degradative changes in protein localization or function [2]. Nowadays, ubiquitination is recognized as a key player in the regulation of a plethora of cellular functions, and its importance for cellular homeostasis is becoming increasingly clear.

The conjugation of ubiquitin to a target protein is a multistep process involving the sequential action of a ubiquitin activating enzyme (E1), a ubiquitin-conjugating enzyme (E2), and a ubiquitin protein-ligase (E3). E3 ligases catalyze the final transfer of ubiquitin to a lysine residue in the target protein, and are largely responsible for the substrate-specificity of the reaction [3]. In some cases, a target protein is modified at one or more lysine residues by the addition of a single ubiquitin molecule (monoubiquitinated). In other cases, the target is decorated with a chain of ubiquitin subunits (polyubiquitinated), linked through one of ubiquitin lysine residues. Since there are seven lysines in the ubiquitin sequence (Lys6, Lys11, Lys27, Lys29, Lys33, Lys48 and Lys63), polyubiquitin chains of different length and topology can be formed [4]. In addition to chains involving internal lysine residues, polyubiquitin chains with a linear topology can also be formed by conjugating multiple ubiquitin subunits through their amino-terminal methionine (Met1) residues, a process catalyzed by a multisubunit E3 ligase complex termed the linear ubiquitin chain assembly complex (LUBAC) [5].

The ubiquitin tags can mediate non-covalent interactions of the ubiquitinated substrate with other proteins bearing different types of ubiquitin-binding motifs. The type of ubiquitin tag and the resulting interactions determine the fate of the substrate [6]. The best-characterized ubiquitin chains are those involving lysine residues Lys48 and Lys63. Lys48 polyubiquitination usually leads to the proteasomal degradation of the substrate. In contrast, monoubiquitination and Lys63 polyubiquitination generally lead to proteasome-independent changes in the localization, in protein-protein interactions, and in activity of the modified protein [7]. The outcome of protein polyubiquitination with chains involving ubiquitin lysines other than Lys48 and Lys63, or the linear polyubiquitination involving Met1, is less well understood, and is the focus of intense research [8].

Ubiquitination is a very common posttranslational modification. Global proteomics analyses have revealed that thousands of cellular proteins are ubiquitinated [9]. Similar to other posttranslational modifications, ubiquitination is a reversible process, and there is a family of enzymes, termed deubiquitinases (DUBs), that act on ubiquitinated substrates to catalyze the removal of ubiquitin moieties [10]. *In vitro* studies have shown that, while some DUBs preferentially cleave specific ubiquitin linkages [11,12], others show a notable promiscuity with respect to the type of ubiquitin linkage they can hydrolyze [13]. Importantly, a DUB that specifically cleaves linear ubiquitin chains has recently been identified [14,15].

Thus, it is the balance between the opposing actions of specific E3 ligases and DUBs which ultimately determines the ubiquitination status of a given target, rendering protein ubiquitination a versatile and dynamic posttranslational modification. The list of cellular processes where ubiquitination plays a regulatory role is continuously expanding, and includes gene expression [16], cell cycle progression [17], apoptosis [18], DNA repair [19] and cell motility [20], among others. Many of these ubiquitination-regulated processes are essential for maintaining cellular homeostasis, and their alteration contributes to tumor development. The importance of ubiquitination in cancer-related aspects of cell function, and the clinical success of the proteasome inhibitor bortezomib in the treatment of multiple myeloma [21] have spurred the interest in the proteins that participate in the processes of ubiquitination/deubiquitination as potential targets for anticancer therapy [22,23]. In this regard, several inhibitors of E3 ligases are currently undergoing clinical trials, as described in detail in recent reviews [23-25]. On the other hand, although a number of DUB inhibitors are being tested in a preclinical setting, the development of DUB-targeted agents is less advanced. This may be due in part to the fact that basic knowledge on DUBs has lagged behind that on E3 ligases. Nevertheless, there has been substantial progress in our understanding of the function and regulation of a subset of DUBs over the last years, and several members of this family are now being actively explored as potential anticancer targets [23,26].

One of the best-characterized DUBs is USP1 (ubiquitin-specific protease 1), which plays an important role in the regulation of DNA repair processes. In this article, we first present a brief overview of the function of human DUBs, emphasizing their increasingly recognized potential as targets in cancer treatment. Then, we focus our attention on USP1, reviewing our current knowledge on the function and regulation of this DUB. Finally, we summarize the alterations of USP1 found in human tumors, and discuss novel findings regarding the potential of this enzyme as a target in the treatment of non-small cell lung cancer (NSCLC), including novel data that extend previously reported findings.

### Potential of human DUBs as novel targets in cancer therapy

The human genome contains around 100 genes encoding for deubiquitinases. Human DUBs can be classified into five different families. Most DUBs are cysteine proteases belonging to one of four families, termed ubiquitin-specific proteases (USPs), ubiquitin carboxy-terminal hydrolases (UCHs), ovarian tumor proteases (OTUs), and Josephins or Machado-Joseph domain (MJD) proteases. A fifth DUB family, termed JAMM/MPN domain-associated metallopeptidases (JAMMs) comprises enzymes with zinc metalloprotease activity [27].

By antagonizing the activity of E3 ligases, DUBs can rescue a ubiquitinated protein from proteasomal degradation, or alter its fate in a more subtle way by editing the length and topology of its ubiquitin tag [28]. DUB activity modulates the ubiquitination state of many cellular proteins, and thus contributes to regulate their levels, activity and localization. Besides these regulatory functions, several DUBs also play a housekeeping function in the maintenance of the cellular pool of free ubiquitin. For example, proteasome-associated DUBs, such as UCHL5, USP14 and POH1, help recycling the ubiquitin chains from proteins before their proteasomal degradation [26].

As comprehensively discussed in recent review articles [29-31], there is mounting evidence supporting the view that human DUBs play an important role in cancer development. Several DUBs involved in cancer-related cellular processes are summarized in Table 1. At least six different DUBs (USP2a, USP4, USP7/HAUSP, USP10, USP29 and USP42) contribute to regulate the crucial tumor suppressor protein p53 [29,32]. On the other hand, the activity of several signalling pathways related to cancer, such as those mediated by EGFR, NFκB, TGFβ or WNT, is, at least in part, controlled by DUBs [30,33]. Regulation of these pathways often involves the concerted action of several DUBs. As an example, EGFR-mediated signal transduction can be regulated at the level of receptor endocytosis, trafficking and degradation by the DUBs USP8/Ubpy, AMSH, USP2a and Cezanne [34-36], but also at a more downstream level by DUBs like USP7/HAUSP or CYLD that modulate the ubiquitination of PTEN or Akt, respectively [37,38]. DUBs may also contribute to the crosstalk between different pathways, as illustrated by USP4, which is regulated by Akt-mediated phosphorylation and deubiquitinates the TGFβ-I receptor [39]. In addition to signal transduction, DUBs also regulate fundamental cellular processes frequently disrupted in cancer, such as cell cycle progression, apoptosis, or the response to DNA damage [29,31,40]. Importantly, altered expression of DUBs seems to be a recurrent finding in different types of human cancer [30].

**Table 1:** Involvement of selected DUBs in the regulation of cancer-related signalling pathways and cellular processes

**DUB**	**Signalling pathway/Process**	**Substrate**
USP2a	Fas and p53	FAS, MDM2, MDMX
	Mitotic progression	Aurora A
	NF-κB	RIP1, TRAF2, TRAF6
	Cell cycle	Cyclin A1
USP4	Wnt	TCF4
	p53	ARF-BP1
	NF-κB	TAK1, TRAF2, TRAF6, RIP1
	TGF-β and AKT	TβRI
	Growth factor receptor pathways	PDK1
USP7	p53	p53, MDM2, MDMX
	Akt	FOXO4, PTEN
USP10	Transcriptional regulation	H2A.Z
	p53	p53
USP29	p53	p53
USP42	p53	p53
USP8/Ubpy	Endosomal sorting	EGFR
	Wg/Wnt	Frizzled
	Hedgehog	Smoothened
AMSH	Endosomal sorting	EGFR
Cezanne	NF-κB	TRAF6, RIP1
CYLD	NF-κB	TRAF2, TRAF6, TRAF7, TAK1, RIP1

Table shows several human DUBs involved in the regulation of different signalling pathways/processes, as well as their reported substrates. An extended version of this Table, including references, is provided as Additional file 1: Table S1.

As a result of all these findings, human DUBs are increasingly regarded a potential targets in cancer therapy [24,41]. Several DUB-targeting compounds have been identified, and are currently being tested in a preclinical setting. Some of these compounds, such as PR-619 and WP1130, inhibit a large subset of DUBs, whereas others, such as b-AP15, exhibit a higher target specificity. PR-619 is a reversible, cell permeable pan-DUB inhibitor that targets a wide range of DUBs, but shows selectivity toward DUBs over other proteases, such as calpain 1, or cathepsins [42]. WP1130, on the other hand, inhibits at least five DUBs: USP5, UCH-L1, USP9X, USP14, and UCH37, and induces the cellular accumulation of polyubiquitinated proteins [43]. Finally, b-AP15 specifically inhibits two proteasome-associated DUBs: UCH-L5 and USP14, and thus blocks proteasome function leading to the accumulation of polyubiquitin in treated cells [44]. PR-619 is being developed mainly as a research tool. With regard to the therapeutic potential of WP1130 and b-AP15, although both compounds have been shown to promote tumor cell apoptosis in preclinical models [43,44], DUB inhibitors with narrower target specificity, such as b-AP15, might be more easily amenable to clinical development, since DUB inhibitors with broader specificity, such as WP1130, may be more prone to cause unwanted side effects that are difficult to predict.

The interest in DUBs as therapeutic targets is further reflected by the impressive number of ongoing efforts to develop and characterize novel DUB inhibitors using innovative strategies, such as chemical synthesis of ubiquitin bioconjugates [45] or phage display-based selection of ubiquitin mutants with enhanced affinity for specific DUBs [46].

An extremely interesting subset of DUBs from the point of view of anticancer therapy are those that function in the cellular response to DNA damage. Many conventional chemotherapeutic drugs, such as cisplatin, are genotoxic agents that may elicit a DNA damage response (DDR). Depending on the type of DNA damage, different DDR pathways are activated that may, in some cases, render cancer cells resistant to chemotherapy [47,48]. DDR pathways are complex multistep processes involving molecular mechanisms for damage recognition, checkpoint activation, signaling, and recruitment of the DNA repair machinery to the site of the lesion. It is becoming increasingly clear that the DDR is critically regulated by ubiquitination and deubiquitination, and thus, the concept has evolved that targeting the enzymes that play a role in these processes, including DUBs [19,49-51], might be an approach to overcome resistance to conventional therapy [40]. It has been suggested, for example, that inhibition of USP7, a DUB that functions in the G2/M checkpoint triggered by DNA damage, might improve treatment efficiency when used in combination with a genotoxic agent [42].

One of the best-characterized human DUBs, USP1, represents a prominent example of a DUB that participates in DDR pathways and may constitute a promising anticancer target.

### Cellular functions and substrates of USP1

**Figure 1:**
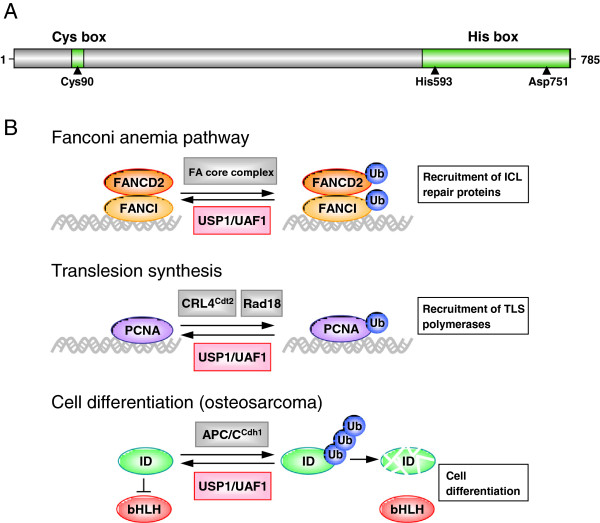
**USP1 Domain structure and summary of the cellular functions of the USP1/UAF1 complex. A.** Shematic representation of USP1 protein showing the position of its amino-terminal Cys box and carboxy-terminal His box domains. The catalytic residues Cys90, His593 and Asp751 are indicated by arrowheads. **B.** Models illustrating the role of the USP1/UAF1 complex as regulator of three different cellular processes. In the Fanconi anemia (FA) pathway (upper panel), the FA core complex monoubiquitinates FANCD2 and FANCI, which mediate the recruitment of other proteins that repair DNA interstrand crosslink (ICL) lesions. USP1/UAF1 antagonizes the FA core complex deubiquitinating FANCD2 and FANCI. In the process of translesion synthesis (TLS) (middle panel), monoubiquitinated PCNA recruits specific TLS DNA polymerases that may bypass DNA lesions, but have lower fidelity than replicative polymerases. USP1/UAF1 may prevent unscheduled recruitment of low fidelity TLS polymerases by deubiquitinating PCNA. Finally (lower panel), USP1/UAF1 contributes to the maintaince of the undifferentiated state of osteosarcoma cells by promoting deubiquitination and stability of ID proteins, which, in turn, negatively regulate differentiation-inducing bHLH transcription factors.

The USP1 gene, cloned in 1998, encodes a 785 amino acid protein with a predicted molecular weight of 88.2 KDa [52]. USP1 bears the conserved USP domain that characterizes this DUB family, with an amino-terminal Cys box motif and a carboxy-terminal His box motif that contain the catalytic residues (Cys90, His593 and Asp751) [52-54] (Figure 1A).

The best-characterized function of USP1 is as a regulator of several important steps in the DNA damage response, mainly in the Fanconi anemia (FA) pathway [55], and in the process of translesion synthesis (TLS) [56]. In addition, recent evidence suggests that USP1 may also contribute to regulate differentiation in specific cellular contexts [57]. As detailed below, these functions are carried out by USP1 in a heterodimeric complex with its cofactor UAF1 (USP1-associated factor 1) [58].

FA is a rare hereditary disorder characterized by congenital abnormalities, progressive bone marrow failure, hypersensitivity to DNA crosslinking agents, genomic instability and increased susceptibility to cancer [59]. Mutations in at least 15 genes have been shown to cause FA. The products of these genes are active in the DNA repair pathway that corrects insterstrand crosslinks (ICL), a DNA lesion that causes polymerase stalling. In response to this type of genomic damage, eight FA proteins assemble in a nuclear complex with ubiquitin E3 ligase activity, termed the FA core complex, which monoubiquitinates two other FA proteins, FANCD2 and FANCI, at the site of the lesion. In turn, monoubiquitinated FANCD2 (ub-FANCD2) and FANCI (ub-FANCI) serve as a platform to recruit the specific nucleases, polymerases and other DNA repair proteins that carry out the subsequent steps of ICL correction (reviewed in [51]). USP1 deubiquitinates both ub-FANCD2 [55] and ub-FANCI [60], thus reverting the critical event in the activation of the FA pathway (Figure 1B, upper panel). USP1-mediated deubiquitination of FANCD2 and FANCI is crucial for the correct function of the FA pathway, as evidenced by the observation that USP1 gene knockout in murine models or DT40 chicken cells recapitulates many phenotypical aspects of FA, including haematopoietic defects and hypersensitivity to DNA crosslinking agents [61-64].

A second DNA repair-related process, translesion synthesis (TLS), is also regulated by USP1, further supporting the crucial role of this DUB in the DNA damage response. The critical USP1 substrate in TLS is PCNA (Proliferating Cell Nuclear Antigen) [56]. Following DNA damage that stalls the progression of the replication fork, PCNA is monoubiquitinated at lysine 164 by the Rad18 E3 ligase [65]. Monoubiquitinated PCNA (ub-PCNA) facilitates the recruitment of specific TLS polymerases, which can bypass the lesion [66]. PCNA can also be monoubiquitinated in the absence of DNA damage by the CRL4^Cdt2^ E3 ubiquitin ligase complex [67]. TLS polymerases have a lower fidelity than replicative polymerases, and may thus result in a higher mutagenesis rate. By reverting PCNA monoubiquitination [56] (Figure 1B, middle panel), USP1 contributes to prevent unscheduled recruitment of TLS polymerases, and may thus help maintaining genome stability [68].

Finally, USP1 has also been reported to contribute to the repair of double-strand DNA breaks through homologous recombination. The molecular mechanism underlying this function is less clear, but it appears to involve suppression of the nonhomologous end-joining pathway [64].

Besides these DNA repair-related functions, USP1 has been recently reported to deubiquitinate and stabilize three members of the family of inhibitors of DNA binding (ID) proteins, namely ID1, ID2 and ID3 [57] (Figure 1B, lower panel). ID proteins are expressed during development in several undifferentiated and proliferating cells [69]. They are negative regulators of basic helix-loop-helix (bHLH) type transcription factors, which bind to DNA and promote the differentiation of various cell types [70]. By deubiquitinating ID proteins, USP1 contributes to prevent bHLH-mediated differentiation, and thus maintain stem-cell characteristics in osteosarcoma cells [57].

### Regulation of USP1 function

Several mechanisms that regulate the expression levels of USP1, its catalytic activity, and its access to substrates have been identified. These mechanisms ensure that USP1 function is carried out in a properly controlled spatio-temporal manner, and include cell cycle-regulated expression, protein-protein interactions, autocleavage/degradation and phosphorylation.

**Figure 2:**
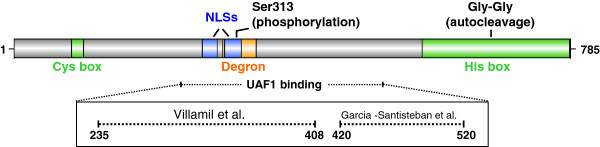
**Regulatory motifs in USP1 amino acid sequence.** Schematic representation of USP1 protein showing the position of sequence motifs that contribute to the regulation of its levels, localization and activity. These regulatory motifs include a destruction box or “degron” (orange) that mediates APC/C^Cdh1^-mediated degradation of USP1, and two NLSs (blue) that mediate import of the USP1/UAF1 complex into the nucleus. The UAF1-binding region of USP1 is still a matter of some controversy. As indicated within the box, two different motifs have been proposed [73,74]. The position of the diglycine motif (Gly-Gly), which constitutes the site of USP1 autocleavage, and the residue Ser313, which is the site of Cdk-mediated USP1 phosphorylation, are also indicated.

Transcription of the USP1 gene is regulated in a cell cycle-dependent manner. USP1 mRNA levels remain low during G1 phase, and reach a peak during S phase [55]. The cell cycle-dependent expression of USP1 is also regulated at the protein level by proteasomal degradation. In this regard, the USP1 region 295-342 (Figure 2) constitutes a destruction motif (degron) that is required for Anaphase Promoting Complex/Cyclosome^Cdh1^ (APC/C^Cdh1^)-dependent degradation of USP1 during G1 [71], which appears to be further modulated by calpains [72].

Arguably, the most critical event in the regulation of USP1 deubiquitinase activity is its interaction with UAF1 [58]. UAF1 is a WD40-repeat containing protein that also binds to and regulates two other DUBs, USP12 and USP46 [75], but its best-characterized role is as a USP1 cofactor in the context of a USP1/UAF1 complex. The enzymatic activity of USP1 alone is very low, and is dramatically increased upon binding to UAF1 [58]. Although the molecular basis for UAF1-mediated USP1 activation remains to be fully understood, recent *in vitro* analyses using artificial substrates support an allosteric mechanism, involving UAF1-induced conformational changes in the USP1 active site [53]. Besides increasing its catalytic activity, UAF1 also stabilizes USP1 [58], and mediates its access to ub-FANCD2 and ub-PCNA [76]. Reaching these nuclear substrates first requires nuclear import of the USP1/UAF1 complex, which is mediated by two nuclear localization signals (NLSs) in USP1 (Figure 2) [73]. Then, a SUMO-like domain (SLD2) in UAF1 mediates the precise targeting of the complex to ub-FANCD2 (through direct binding to FANCI) [76] and ub-PCNA (through direct binding to the PCNA partner ATAD5/ELG1) [77]. Thus, targeting of USP1/UAF1 to FANCD2 and PCNA can be regarded as a two-step process that requires specific sequence motifs in both partners. Intriguingly, a short sequence motif in USP1 (residues 141-159) has been shown to function as a nuclear export signal (NES) when tested in a nuclear export assay [78]. However, the functional relevance of this motif in the context of full-length USP1 remains to be established.

The USP1-binding region in UAF1 is relatively large, and includes its eight predicted WD40 repeats [58]. The identity of the UAF1-binding region in USP1, on the other hand, is still a matter of some controversy (Figure 2). By using a cellular relocation assay and co-immunoprecipitation with GFP-tagged USP1, the UAF1-binding site was reported to comprise USP1 region 420-520 [73]. In contrast, by using pull-down assays with bacterially expressed GST-tagged proteins, the UAF1-binding site was mapped to USP1 region 235-408 [74]. It is still unclear which one of these USP1 motifs mediates UAF1 binding, and it remains also possible that more than one amino acid segment in USP1 contribute to the interaction with UAF1. Further experiments and, eventually, the solution of USP1 three-dimensional structure should clarify this issue.

Besides the cell cycle-dependent expression of USP1 described above, there are mechanisms that facilitate inducible changes in the levels and activity of USP1 in response to genotoxic insults, as expected from its DNA damage-related functions. Activation of the FA or TLS pathways would require down-regulation of USP1 to prevent deubiquitination of FANCD2/FANCI and PCNA. In this regard, transcription of USP1 gene is halted after DNA damage [58], by a mechanism involving the p21 cyclin-dependent kinase inhibitor [79]. On the other hand, USP1 protein levels are downregulated in cells exposed to genotoxic agents, such as UV, by a mechanism that involves autocleavage at an internal diglycine motif (Gly670-Gly671) (Figure 2) [56]. USP1 autocleavage results in the generation of an amino-terminal fragment (USP1^NT^) and a shorter carboxy-terminal fragment (USP1^CT^). Autocleaved USP1 remains enzymatically active [58] and thus, effective USP1 downregulation requires subsequent proteasomal degradation of the fragments [56] that, in the case of USP1^CT^, is mediated by the N-end rule pathway [80].

The observation that autocleaved USP1 remains active was unexpected, since the cleavage event disrupts its His box catalytic motif. In this regard, it has been proposed that USP1^NT^ and USP1^CT^ fragments may be held together by UAF1 forming a catalytically competent ternary complex [58]. This model, however, seems difficult to reconcile with the finding that the carboxy-terminal region of USP1 is unable to interact with UAF1 [73], and would require further experimental validation.

In contrast to the irreversible destruction of USP1 triggered by UV exposure, reactive oxigen species (ROS) have recently been shown to induce a reversible inactivation of USP1 and also of other cysteine protease DUBs [81-83]. Mechanistically, inactivation of DUBs by ROS results from the oxidation of their catalytic cysteine residue, and the sensitivity of these enzymes to oxidative inhibition is associated with their activation, which, in the case of USP1, requires its interaction with UAF1 [81,82]. These findings uncover a novel role for DUBs as mediators of the cellular response to oxidative stress. In particular, ROS-mediated regulation of USP1/UAF1 activity would contribute to modulate the level of PCNA monoubiquitination in response to oxidative stress [81,82].

Another reversible modification of USP1 is phosphorylation at Ser313 by cyclin-dependent kinases (CDKs) [84]. Two functional consequences of Ser313 phosphorylation have been reported. On one hand, phosphorylation of this residue, which is located within USP1 degron motif (Figure 2), may contribute to regulate the cell-cycle dependent levels of USP1, by preventing its degradation in mitosis [84]. On the other hand, phosphorylation of Ser313 has been reported to be necessary for USP1 interaction with UAF1 in an *in vitro* experimental setting [74], thus raising the possibility that Ser313 phosphorylation could be a critical regulatory event for USP1 enzymatic activity, although these findings await further confirmation in a more physiological setting.

### USP1 alteration in cancer

**Figure 3:**
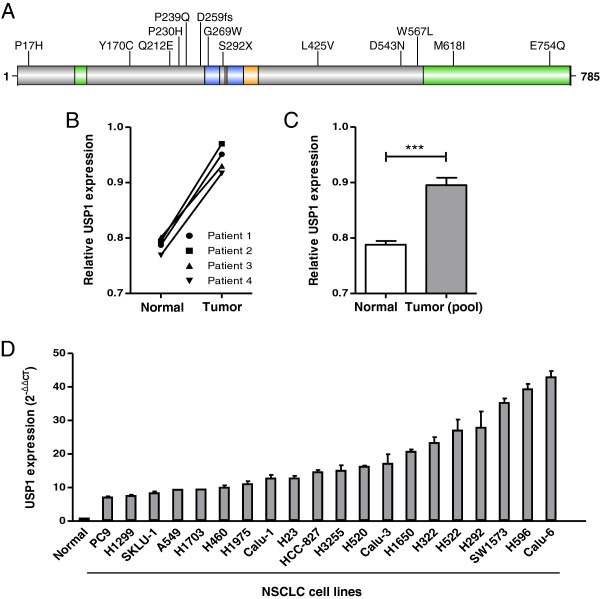
**USP1 gene mutations and altered USP1 mRNA expression in lung cancer. A.** Shematic representation of USP1 protein showing the position of 13 lung cancer-associated USP1 mutations reported to date (5 April 2013) in the COSMIC mutation database. Amino acid changes are indicated using the one-letter code. **B.** Relative USP1 mRNA expression normalized to GAPDH in paired samples of tumor tissue and adjacent normal tissue from four NSCLC patients (represented by different symbols), showing higher USP1 expression in tumor tissue. Total RNA was isolated using Trizol (Invitrogen), and complementary DNA (cDNA) was synthesized using the Dynamo cDNA synthesis kit for qRT-PCR (Thermo Scientific). Quantitative real-time PCR (qRT-PCR) was performed using the TaqMan Universal Master Mix (Applied Biosystems) according to the manufacturer’s instructions on an ABI Prism 7500 instrument. Assay-on-Demand Gene expression primers and probes (Applied Biosystems) were used to specifically amplify USP1 (Hs00163427_m1) and Human GAPD (GAPDH) Endogenous Control (4326317E). **C.** Graph comparing the relative expression of USP1 mRNA normalized to GAPDH internal control in normal lung tissue (white bar; four individual samples) with the expression in pooled samples of the three different NSCLC histological subtypes (grey bar; adenocarcinoma, squamous cell carcinoma and large cell carcinoma). Each pooled sample included laser-microdissected specimens from five different patients, as described previously [88]. qRT-PCR analysis was carried out as described above. Graph bars show mean ± SEM. USP1 was significantly overexpressed in the pooled tumor samples (***P < 0.001). **D.** Relative USP1 mRNA expression normalized to GAPDH in 20 different NSCLC cell lines compared to the average of four normal lung tissue mRNA samples. qRT-PCR analysis was carried out as described above. The relative mRNA levels were determined with the ΔΔCt method using the “normal” value as a reference. Graph bars show mean ± SD of two replicates.

Mutational alteration of human DUBs, including USP1, does not appear to be a frequent event in cancer [30]. With the exception of CYLD, mutated in the familial cylindromatosis syndrome [85], and BAP1, mutated in several malignancies [86], no other DUBs have been reported to be recurrently mutated in specific tumor types. Nevertheless, whole genome analysis of cancer samples is uncovering low-frequency DUB mutations in human tumors. In the case of USP1, a survey of the COSMIC mutation database [87] revealed a total of 23 non-synonymous mutations in different tumor types. Thirteen of these mutations have been detected in lung cancer samples. Figure 3A shows the distribution of these lung cancer-associated mutations on USP1 protein. Most of these are missense mutations, resulting in amino acid substitutions, but the functional consequences of these protein changes remain to be established.

Contrasting with the low prevalence of DUB gene mutations, altered mRNA expression of human DUBs is a recurrent finding in cancer [30,89]. In this regard, a survey of publically available cancer microarray expression data using the Oncomine Research Edition database [90] reveals that USP1 mRNA expression has been found to be significantly altered (Fold change > 1.5; P-value < 0.05) in several tumor types (Table 2). Aberrant overexpression is the most common finding, and it appears to be particularly frequent in cervical and gastric cancer, melanoma and sarcoma, four tumor types where USP1 results overexpressed in more than 60% of the available studies. Interestingly, a more focused study of USP1 expression in osteosarcoma samples demonstrated aberrantly high levels of both USP1 mRNA and protein, which correlated with increased expression of its substrate ID2 [57].

**Table 2:** USP1 over/underexpression in several cancer tissue types

**Cancer type**	**Overexpressed**	**Underexpressed**
Sarcoma	11/15 (73%)	0/15 (0%)
Melanoma	4/6 (67%)	1/6 (17%)
Gastric cancer	5/8 (63%)	0/8 (0%)
Cervical cancer	3/5 (60%)	0/5 (0%)
Brain and CNS cancer	10/20 (50%)	1/20 (5%)
Liver cancer	3/10 (30%)	0/10 (0%)
Other cancer	8/27 (30%)	6/27 (22%)
Lung cancer	4/16 (25%)	0/16 (0%)
Bladder cancer	2/9 (22%)	0/9 (0%)
Myeloma	1/5 (20%)	0/5 (0%)
Colorectal cancer	5/26 (19%)	0/26 (0%)
Head and neck cancer	4/25 (16%)	0/25 (0%)
Esophageal cancer	1/10 (10%)	0/10 (0%)
Kidney cancer	2/20 (10%)	0/20 (0%)
Ovarian cancer	1/11 (9%)	1/11 (9%)
Lymphoma	2/27 (7%)	3/27 (11%)
Breast cancer	1/31 (3%)	1/31 (3%)
Leukemia	0/22 (0%)	4/22 (18%)
Pancreatic cancer	0/8 (0%)	0/8 (0%)
Prostate cancer	0/16 (0%)	1/16 (6%)

The publicly avalilable Oncomine cancer microarray database (accessed in April 2013) was queried to systematically assess USP1 expression levels across different cancer types versus the corresponding normal tissue (Analysis type: Cancer vs Normal). In the “Gene summary view” page, the following thresholds were set: P-value < 0.05; Fold-change > 1.5; Gene-rank, Top 10%. As “Data-type”, mRNA was selected. For each cancer type, the number of analyses that met the thresholds for overexpression or underexpression was recorded, and is shown in the Table as a ratio (and percentage) with respect to the total number of analyses in the database.

Current data on USP1 expression in lung cancer are somewhat controversial. USP1 overexpression (Fold change > 1.5; P-value < 0.05) was reported in 25% (4/16) of the lung cancer microarray sets available through Oncomine, whereas none of these studies reported significant downregulation of USP1. In line with these findings, USP1 protein overexpression has been found by immunohistochemical analysis on a NSCLC tissue microarray containing 90 paired samples [91]. In contrast, a study using quantitative RT-PCR and western blot analysis reported down-regulation of USP1 mRNA and protein in NSCLC with respect to adjacent tissue [92]. In an attempt to further clarify this issue, we used quantitative RT-PCR to analyze USP1 mRNA levels in paired normal/tumor tissue samples from four NSCLC patients (Figure 3B). We also compared USP1 expression in normal lung tissue *versus* three pooled mRNA samples of different NSCLC histological subtypes (adenocarcinoma, squamous cell carcinoma and large cell carcinoma), each including five individual patients (Figure 3C). Finally, we measured USP1 mRNA levels in a panel of 20 NSCLC cell lines (Figure 3D). In all these settings, USP1 expression was higher in tumors or tumor-derived cells than in normal samples, supporting the association of USP1 overexpression with NSCLC.

### Emerging potential of USP1 as a novel target in NSCLC

Many commonly used chemotherapeutic drugs induce DNA damage, and chemoresistance may arise if cancer cells acquire an increased ability to repair or tolerate DNA lesions [93]. The important regulatory role of USP1 in DNA repair, supported by the finding that USP1 gene knockout in model systems leads to DNA damage hypersensitivity [62-64], together with the observation that USP1 is frequently overexpressed in tumors, suggests that USP1 could be a relevant target for cancer therapy, whose inhibition might contribute to overcome chemoresistance.

This view has recently gained experimental support with the identification of two compounds that inhibit the activity of the USP1/UAF1 complex, and reverse the resistance of NSCLC cells to cisplatin [94], a DNA damaging drug commonly used in cancer chemotherapy. In this study, a high-throughput screening of nearly 10,000 bioactive small-molecules, followed by further validation and characterization of the best candidates, led to the identification of two potent and highly selective reversible inhibitors of the enzymatic activity of the USP1/UAF1 complex, pimozide and GW7647. These inhibitors were subsequently tested in two NSCLC cell lines to evaluate their cytotoxic activity in combination with cisplatin. Using cell viability assays and determination of the combination index (C.I.) [95,96], pimozide and GW7647 showed synergistic activity with cisplatin in a cisplatin-resistant NSCLC cell line (H596), but not in a cisplatin-sensitive cell line (H460).

**Figure 4:**
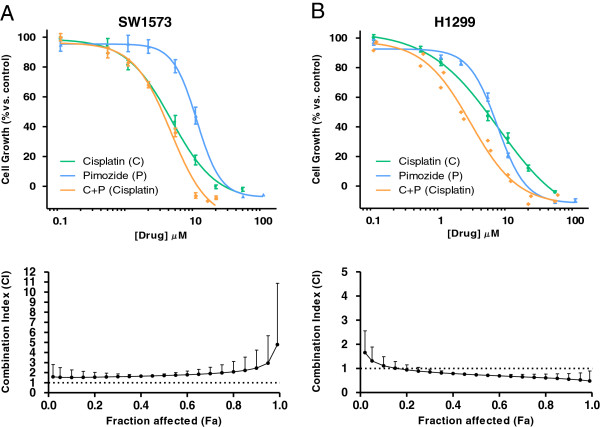
**Growth inhibition curves and median-drug effect plots of two NSCLC cell lines treated with cisplatin, pimozide or their simultaneous combination.** Exponentially growing SW1573 **(A)** and H1299 **(B)** NSCLC cells were seeded in 96-well plates. Twenty four hours after seeding, cells were treated with cisplatin, pimozide or a combination of both drugs. After 72 hours of drug exposure, the sulforhodamine B (SRB) chemosensitivity assay [97] was performed. Briefly, cells were fixed with ice-cold trichloroacetic acid, washed with deionized water, left to dry and stained with SRB dye. After washing with 1% acetic acid, cells were left to dry, and protein-bound dye was dissolved in 10 mM Tris base solution. OD at 492 nm was determined. Growth inhibition curves (upper panels) were generated, where each point represents mean ± SEM of at least 3 replicates. IC_50_ values, the drug concentration that inhibits the cell growth by 50% (summarized in Table 3), were calculated by fitting the data to a sigmoid dose–response curve using GraphPad Prism. The treatment with the two drugs in combination was carried out using a constant cisplatin:pimozide ratio (as indicated in Table 3), which was established for each cell line as the ratio of the IC_50_ values for each single drug. The drug concentrations on the X-axis of the growth inhibition curves for the combination treatment refer to cisplatin. In order to evaluate the interaction between cisplatin and pimozide we used the median-drug effect analysis method originally described by Chou and Talalay [95]. Using CalcuSyn, the effect of the combination was compared to the effect of each drug to determine the combination index (C.I.) at a range of Fa (fraction affected by the dose) values. The resulting C.I. values were plotted against the Fa to produce the median-drug effect plot for each cell line (lower panels).

In an attempt to extend these findings to a larger number of NSCLC cell lines, we tested the effect of pimozide in combination with cisplatin on a panel of seven NSCLC cell lines. A cell viability assay was used to determine the cytotoxic activity of cisplatin, pimozide or a combination of both agents. Figure 4 shows representative examples of the results of this assay in two NSCLC cell lines, and the data for the remaining cell lines analyzed are shown in Additional file 2: Figure S1. The C.I. was subsequently calculated using Calcusyn. The cell lines tested displayed a wide range of cisplatin sensitivity, as summarized in Table 3, and were classified as “ciplatin-sensitive” if their IC_50_ was below the mean IC_50_ of the panel (6.2 μM), or as “cisplatin-resistant” if their IC_50_ was above the mean. Pimozide showed a synergistic or additive effect with cisplatin in the three “resistant” cell lines (H1299, H1703 and H520). In contrast, the effect of the pimozide/cisplatin combination was antagonistic in three of the four “sensitive” cell lines (H322, SKLU-1 and SW1573). These findings are consistent with those of Chen et al. [94], and suggest that USP1 inhibition may contribute to revert cisplatin resistance in some preclinical models of NSCLC.

**Table 3:** Effect of the combination between the USP1/UAF1 inhibitor pimozide with the chemotherapy agent cisplatin in a panel of NSCLC cell lines

**Cell line**	**Cisplatin**	**Pimozide**	**Ratio C:P**	**C.I.**	**Combination effect**
	**IC**_**50 **_**(μM)**	**IC**_**50 **_**(μM)**			
*H322 (s)*	1.6	11.6	1:7	1.3	Antagonistic
*SKLU-1 (s)*	2.4	13.7	1:6	1.2	Antagonistic
*SW1573 (s)*	4.4	10.5	1:2	2.0	Antagonistic
*H522 (s)*	5.0	10.8	1:2	0.9	Additive
*H1299 (r)*	7.5	7.0	1:1	0.6	Synergistic
*H1703 (r)*	8.8	5.0	2:1	1.0	Additive
*H520 (r)*	13.7	8.7	2:1	1.1	Additive

The IC_50_ value for cisplatin and pimozide was calculated for each cell line as described in Figure 4. Cell lines were classified as “cisplatin-sensitive” (s) if their IC_50_ for this drug was below the mean value of the panel (6.2 μM), or as “cisplatin-resistant” (r) if their IC_50_ was above the mean. Treatment with the two drugs in combination was carried out using a constant ratio cisplatin:pimozide (Ratio C:P) that was individually established for each cell line as the ratio of the IC_50_ values for each single drug. The combination index (C.I) value was calculated at a full range of Fa values using CalcuSyn, as described in Figure 4. We calculated the final C.I. value for each experiment as the average C.I. at Fa = 0.5, 0.75 and 0.9. At least three independent experiments per cell line were carried out, and the C.I. value indicated in the Table represents the mean of the final C.I. values of these experiments. According to the C.I., the nature of the interaction between the drugs (Combination effect) was classified as synergistic (C.I. < 0.8), additive (0.8 < C.I. < 1.2) or antagonistic (C.I. > 1.2).

### Conclusions and future directions

Although significant advances have been made in recent years, more work is required to achieve a better understanding of the basic mechanisms of USP1 function and regulation. Characterizing the full set of protein-protein interactions involving USP1 (the “USP1 interactome”) would be a significant step in this direction. In this regard, a comprehensive proteomic analysis [98] has identified a set of 23 novel high confidence candidate USP1-interacting proteins. Besides UAF1, this set includes, among others, three other DUBs of the USP family (USP3, USP4 and USP11) and the phosphatase PHLPP. The findings of this global study provide a starting point for more detailed investigations of each specific interaction, which may lead to the identification of novel substrates and regulators of USP1.

Regarding its potential as a target for cancer therapy, the inhibition of USP1 as a strategy to modulate the efficacy of cisplatin and other DNA damaging drugs warrants further investigation. For example, the cellular factors that determine the effect of the combination of USP1 inhibitors with cisplatin need to be elucidated. On the other hand, the recent finding that USP1 contributes to block differentiation in osteosarcoma [57] raises the possibility that USP1 inhibition could be explored as a strategy for differentiation therapy in this tumor type.

Besides inhibiting its enzymatic activity, an alternative approach to target USP1 could be interfering with the formation of the USP1/UAF1 complex, which is not disrupted by pimozide and GW7647 [94]. In this regard, a relocation-based assay for USP1/UAF1 binding, which could be used to screen for compounds that disrupt their interaction, has been recently described [73]. This approach would greatly benefit from a knowledge of the three-dimensional structure of the USP1/UAF1 complex, and from a clarification of the controversy regarding the USP1 region that mediates UAF1 binding [73,74].

## Abbreviations

APC/CCdh1: Anaphase-promoting complex/cyclosome; bHLH: Basic helix-loop-helix; C.I.: Combination index; DDR: DNA damage response; DUB: Deubiquitinase; FA: Fanconi anemia; Fa: Fraction affected by the dose; GST: Glutathione S-transferase; IC50: Half maximal inhibitory concentration; ICL: Interstrand crosslink; ID: Inhibitor of DNA binding; JAMM: JAMM/MPN domain-associated metallopeptidase; LUBAC: Linear ubiquitin chain assembly complex; MJD: Machado-Joseph domain; NES: Nuclear export signal; NLS: Nuclear localization signal; NSCLC: Non-small cell lung cancer; OTU: Ovarian tumor protease; PCNA: Proliferating cell nuclear antigen; ROS: Reactive oxigen species; SRB: Sulforhodamine B; TLS: Translesion synthesis; UAF1: USP1-associated factor 1; UCH: Ubiquitin carboxy-terminal hydrolase; USP1: Ubiquitin-specific protease 1; UV: Ultraviolet.

## Competing interests

The authors declare that they have no competing interests.

## Authors’ contributions

IG-S carried out analyses, discussed data, and drafted the article. GJP discussed data and contributed to writing the article. EG discussed data, and contributed to writing the article. JAR conceived study, discussed data, and contributed to writing the article. All authors read and approved the final manuscript.

## Supplementary Material

Additional file 1: Table S1 Involvement of selected DUBs in the regulation of cancer-related signalling pathways and cellular processes. Table shows several shuman DUBs involved in the regulation of different signalling pathways/processes, as well as their reported substrates. Click here for file

Additional file 2: Figure S1 Growth inhibition curves and the corresponding median-drug effect plots of five NSCLC cell lines treated with cisplatin, pimozide or their simultaneous combination. Exponentially growing NSCLC cells were treated and processed as described in Figure 4. Growth inhibition curves (left panels) were generated, where each point represents mean ± SEM of at least 3 replicates. IC50 values, the drug concentration that inhibits the cell growth by 50% (summarized in Table 3), were calculated by fitting the data to a sigmoid dose–response curve using GraphPad Prism. The treatment with the two drugs in combination was carried out using a constant ratio cisplatin:pimozide (as indicated in Table 3), which was established for each cell line as the ratio of the IC50 values for each single drug. The drug concentrations on the X-axis of the growth inhibition curves for the combination treatment refer to cisplatin. In order to evaluate the interaction between cisplatin and pimozide we used the median-drug effect analysis method originally described by Chou and Talalay. Using CalcuSyn software, the effect of the combination was compared to the effect of each drug to determine the combination index (C.I.) at a range of Fa (fraction affected by the dose) values. The resulting C.I. values were plotted against the Fa to produce the median-drug effect plot for each cell line (right panels). Click here for file
