# Supplementary material for: USP1 deubiquitinase: cellular functions, regulatory mechanisms and emerging potential as target in cancer therapy
Source: Mol Cancer. 2013 Aug 10;12:91. doi: 10.1186/1476-4598-12-91 (PMC3750636; doi:10.1186/1476-4598-12-91)
Supplement: Additional file 1: Table S1 — Involvement of selected DUBs in the regulation of cancer-related signalling pathways and cellular processes. Table shows several shuman DUBs involved in the regulation of different signalling pathways/processes, as well as their reported substrates. [file 1476-4598-12-91-S1.pdf]

Supplementary Table 1. Involvement of selected DUBs in the regulation of cancer-related signalling pathways and cellular processes. Table shows several human DUBs involved in the regulation of different signalling pathways/processes, as well as their reported substrates.

DUB	Signalling pathway/Process	Substrate	Reference
USP2a	Fas and p53	FAS	*Graner et al., 2004*
		MDM2	*Stevenson et al., 2007*
		MDMX	*Allende-Vega et al., 2010*
	Mitotic progression	Aurora A	*Shi et al., 2011*
	NF-κB	RIP1, TRAF2	*Mahul-Mellier et al., 2012*
		TRAF6	*He et al., 2013*
	Cell cycle	Cyclin A1	*Kim et al., 2012*
USP4	Wnt	TCF4	*Zhao et al., 2009*
	p53	ARF-BP1	*Zhang et al., 2011*
	NF-κB	TAK1	*Fan et al., 2011*
		TRAF2, TRAF6	*Xiao et al., 2012*
		RIP1	*Hou et al., 2013*
	TGF-β and AKT	TβRI	*Zhang et al., 2012*
	Akt and other central pathways	PDK1	*Uras et al., 2012*
USP7	p53	p53	*Li et al., 2002*
		MDM2	*Li et al., 2004*
		MDMX	*Meulmeester et al., 2005*
	Akt	FOXO4	*van der Horst et al., 2006*
		PTEN	*Song et al., 2008*
USP10	Transcriptional regulation	H2A.Z	*Draker et al., 2011*
	p53	p53	*Yuan et al., 2010*
USP29	p53	p53	*Liu et al., 2011*
USP42	p53	p53	*Hock et al., 2011*
USP8/Ubpy	Endosomal sorting	EGFR	*Mizuno et al., 2005*
	Wg/Wnt	Frizzled	*Mukai et al., 2010*
	Hedgehog	Smoothened	*Xia et al., 2012*
AMSH	Endosomal sorting	EGFR	*McCullough et al., 2004*
Cezanne	NF-κB	TRAF6	*Evans et al., 2001*
		RIP1	*Enesa et al., 2008*
CYLD	NF-κB	TRAF2, TRAF6	*Trompouki et al., 2003*
		TRAF6, TRAF7	*Yoshida et al., 2005*
		TAK1	*Reiley et al., 2007*
		RIP1	*Wright et al., 2007*

Graner E, Tang D, Rossi S, Baron A, Migita T, Weinstein LJ, Lechpammer M, Huesken D, Zimmermann J, Signoretti S, Loda M: **The isopeptidase USP2a regulates the stability of fatty acid synthase in prostate cancer.** *Cancer Cell* 2004, **5**(3):253-261.

Stevenson LF, Sparks A, Allende-Vega N, Xirodimas DP, Lane DP, Saville MK: **The deubiquitinating enzyme USP2a regulates the p53 pathway by targeting Mdm2.** *EMBO J* 2007, **26**(4):976-986.

Allende-Vega N, Sparks A, Lane DP, Saville MK: **MdmX is a substrate for the deubiquitinating enzyme USP2a.** *Oncogene* 2010, **29**(3):432-441.

Shi Y, Solomon LR, Pereda-Lopez A, Giranda VL, Luo Y, Johnson EF, Shoemaker AR, Leverson J, Liu X: **Ubiquitin-specific cysteine protease 2a (USP2a) regulates the stability of Aurora-A.** *J Biol Chem* 2011, **286**(45):38960-38968.

Mahul-Mellier AL, Pazarentzos E, Datler C, Iwasawa R, AbuAli G, Lin B, Grimm S: **De-ubiquitinating protease USP2a targets RIP1 and TRAF2 to mediate cell death by TNF.** *Cell Death Differ* 2012, **19**(5):891-899.

He X, Li Y, Li C, Liu LJ, Zhang XD, Liu Y, Shu HB: **USP2a negatively regulates IL-1β- and virus-induced NF-κB activation by deubiquitinating TRAF6.** *J Mol Cell* Biol 2013, **5**(1):39-47.

Kim J, Kim WJ, Liu Z, Loda M, Freeman MR: **The ubiquitin-specific protease USP2a enhances tumor progression by targeting cyclin A1 in bladder cancer.** *Cell Cycle* 2012, **11**(6):1123-1130.

Zhao B, Schlesiger C, Masucci MG, Lindsten K: **The ubiquitin specific protease 4 (USP4) is a new player in the Wnt signalling pathway.** J Cell Mol Med 2009, **13**(8B):1886-1895.

Zhang X, Berger FG, Yang J, Lu X: **USP4 inhibits p53 through deubiquitinating and stabilizing ARF-BP1.** *EMBO J* 2011, **30**(11):2177-2189.

Fan YH, Yu Y, Mao RF, Tan XJ, Xu GF, Zhang H, Lu XB, Fu SB, Yang J: **USP4 targets TAK1 to downregulate TNFα-induced NF-κB activation.** *Cell Death Differ* 2011, **18**(10):1547-1560.

Xiao N, Li H, Luo J, Wang R, Chen H, Chen J, Wang P: **Ubiquitin-specific protease 4 (USP4) targets TRAF2 and TRAF6 for deubiquitination and inhibits TNFα-induced cancer cell migration.** *Biochem J* 2012, **441**(3):979-986.

Hou X, Wang L, Zhang L, Pan X, Zhao W: **Ubiquitin-specific protease 4 promotes TNF-α-induced apoptosis by deubiquitination of RIP1 in head and neck squamous cell carcinoma.** *FEBS Lett* 2013, **587**(4):311-316.

Zhang L, Zhou F, Drabsch Y, Gao R, Snaar-Jagalska BE, Mickanin C, Huang H, Sheppard KA, Porter JA, Lu CX, ten Dijke P: **USP4 is regulated by AKT phosphorylation and directly deubiquitylates TGF-β type I receptor.** *Nat Cell Biol* 2012, **14**(7):717-726.

Uras IZ, List T, Nijman SM: **Ubiquitin-specific protease 4 inhibits mono-ubiquitination of the master growth factor signaling kinase PDK1.** PLoS One 2012, **7**(2):e31003.

Li M, Chen D, Shiloh A, Luo J, Nikolaev AY, Qin J, Gu W: **Deubiquitination of p53 by HAUSP is an important pathway for p53 stabilization.** Nature 2002, **416**(6881):648-653.

Li M, Brooks CL, Kon N, Gu W: **A dynamic role of HAUSP in the p53-Mdm2 pathway.** Mol Cell. 2004, **13**(6):879-886.

Meulmeester E, Maurice MM, Boutell C, Teunisse AF, Ovaa H, Abraham TE, Dirks RW, Jochemsen AG: **Loss of HAUSP-mediated deubiquitination contributes to DNA damage-induced destabilization of Hdmx and Hdm2.** *Mol Cell* 2005, **18**(5):565-576.

van der Horst A, de Vries-Smits AM, Brenkman AB, van Triest MH, van den Broek N, Colland F, Maurice MM, Burgering BM: **FOXO4 transcriptional activity is regulated by monoubiquitination and USP7/HAUSP.** *Nat Cell Biol* 2006, **8**(10):1064-1073.

Song MS, Salmena L, Carracedo A, Egia A, Lo-Coco F, Teruya-Feldstein J, Pandolfi PP: **The deubiquitinylation and localization of PTEN are regulated by a HAUSP-PML network.** *Nature* 2008, **455**(7214):813-817.

Draker R, Sarcinella E, Cheung P. **USP10 deubiquitylates the histone variant H2A.Z and both are required for androgen receptor-mediated gene activation.** *Nucleic Acids Res* 2011, **39**(9):3529-3542.

Yuan J, Luo K, Zhang L, Cheville JC, Lou Z: **USP10 regulates p53 localization and stability by deubiquitinating p53.** *Cell* 2010, **140**(3):384-396.

Liu J, Chung HJ, Vogt M, Jin Y, Malide D, He L, Dundr M, Levens D: **JTV1 co-activates FBP to induce USP29 transcription and stabilize p53 in response to oxidative stress.** *EMBO J* 2011, **30**(5):846-858.

Hock AK, Vigneron AM, Carter S, Ludwig RL, Vousden KH: **Regulation of p53 stability and function by the deubiquitinating enzyme USP42.** *EMBO J* 2011, **30**(24):4921-4930.

Mizuno E, Iura T, Mukai A, Yoshimori T, Kitamura N, Komada M: **Regulation of epidermal growth factor receptor down-regulation by UBPY-mediated deubiquitination at endosomes.** *Mol Biol Cell* 2005, **16**(11):5163-5174.

Mukai A, Yamamoto-Hino M, Awano W, Watanabe W, Komada M, Goto S: **Balanced ubiquitylation and deubiquitylation of Frizzled regulate cellular responsiveness to Wg/Wnt.** *EMBO J* 2010, **29**(13):2114-2125.

Xia R, Jia H, Fan J, Liu Y, Jia J: **USP8 promotes smoothened signaling by preventing its ubiquitination and changing its subcellular localization.** *PLoS Biol* 2012, **10**(1):e1001238.

McCullough J, Clague MJ, Urbé S: **AMSH is an endosome-associated ubiquitin isopeptidase.** *J Cell Biol* 2004, **166**(4):487-492.

Evans PC, Taylor ER, Coadwell J, Heyninck K, Beyaert R, Kilshaw PJ: **Isolation and characterization of two novel A20-like proteins.** *Biochem J* 2001, **357**(Pt 3):617-623.

Enesa K, Zakkar M, Chaudhury H, Luong le A, Rawlinson L, Mason JC, Haskard DO, Dean JL, Evans PC: **NF-kappaB suppression by the deubiquitinating enzyme Cezanne: a novel negative feedback loop in pro-inflammatory signaling.** *J Biol Chem* 2008, **283**(11):7036-7045.

Trompouki E, Hatzivassiliou E, Tsichritzis T, Farmer H, Ashworth A, Mosialos G: **CYLD is a deubiquitinating enzyme that negatively regulates NF-kappaB activation by TNFR family members.** *Nature* 2003, **424**(6950):793-796.

Yoshida H, Jono H, Kai H, Li JD: **The tumor suppressor cylindromatosis (CYLD) acts as a negative regulator for toll-like receptor 2 signaling via negative cross-talk with TRAF6 AND TRAF7.** *J Biol Chem* 2005, **280**(49):41111-41121.

Reiley WW, Jin W, Lee AJ, Wright A, Wu X, Tewalt EF, Leonard TO, Norbury CC, Fitzpatrick L, Zhang M, Sun SC: **Deubiquitinating enzyme CYLD negatively regulates the ubiquitin-dependent kinase Tak1 and prevents abnormal T cell responses.** *J Exp Med* 2007, **204**(6):1475-1485.

Wright A, Reiley WW, Chang M, Jin W, Lee AJ, Zhang M, Sun SC: **Regulation of early wave of germ cell apoptosis and spermatogenesis by deubiquitinating enzyme CYLD.** *Dev Cell* 2007, **13**(5):705-716.
