# Supplementary material for: USP1 deubiquitinase: cellular functions, regulatory mechanisms and emerging potential as target in cancer therapy
Source: Mol Cancer. 2013 Aug 10;12:91. doi: 10.1186/1476-4598-12-91 (PMC3750636; doi:10.1186/1476-4598-12-91)
Supplement: Additional file 2: Figure S1 — Growth inhibition curves and the corresponding median-drug effect plots of five NSCLC cell lines treated with cisplatin, pimozide or their simultaneous combination. Exponentially growing NSCLC cells were treated and processed as described in Figure 4. Growth inhibition curves (left panels) were generated, where each point represents mean ± SEM of at least 3 replicates. IC50 values, the drug concentration that inhibits the cell growth by 50% (summarized in Table 3), were calculated by fitting the data to a sigmoid dose–response curve using GraphPad Prism. The treatment with the two drugs in combination was carried out using a constant ratio cisplatin:pimozide (as indicated in Table 3), which was established for each cell line as the ratio of the IC50 values for each single drug. The drug concentrations on the X-axis of the growth inhibition curves for the combination treatment refer to cisplatin. In order to evaluate the interaction between cisplatin and pimozide we used the median-drug effect analysis method originally described by Chou and Talalay. Using CalcuSyn software, the effect of the combination was compared to the effect of each drug to determine the combination index (C.I.) at a range of Fa (fraction affected by the dose) values. The resulting C.I. values were plotted against the Fa to produce the median-drug effect plot for each cell line (right panels). [file 1476-4598-12-91-S2.pdf]

Supplementary Figure 1

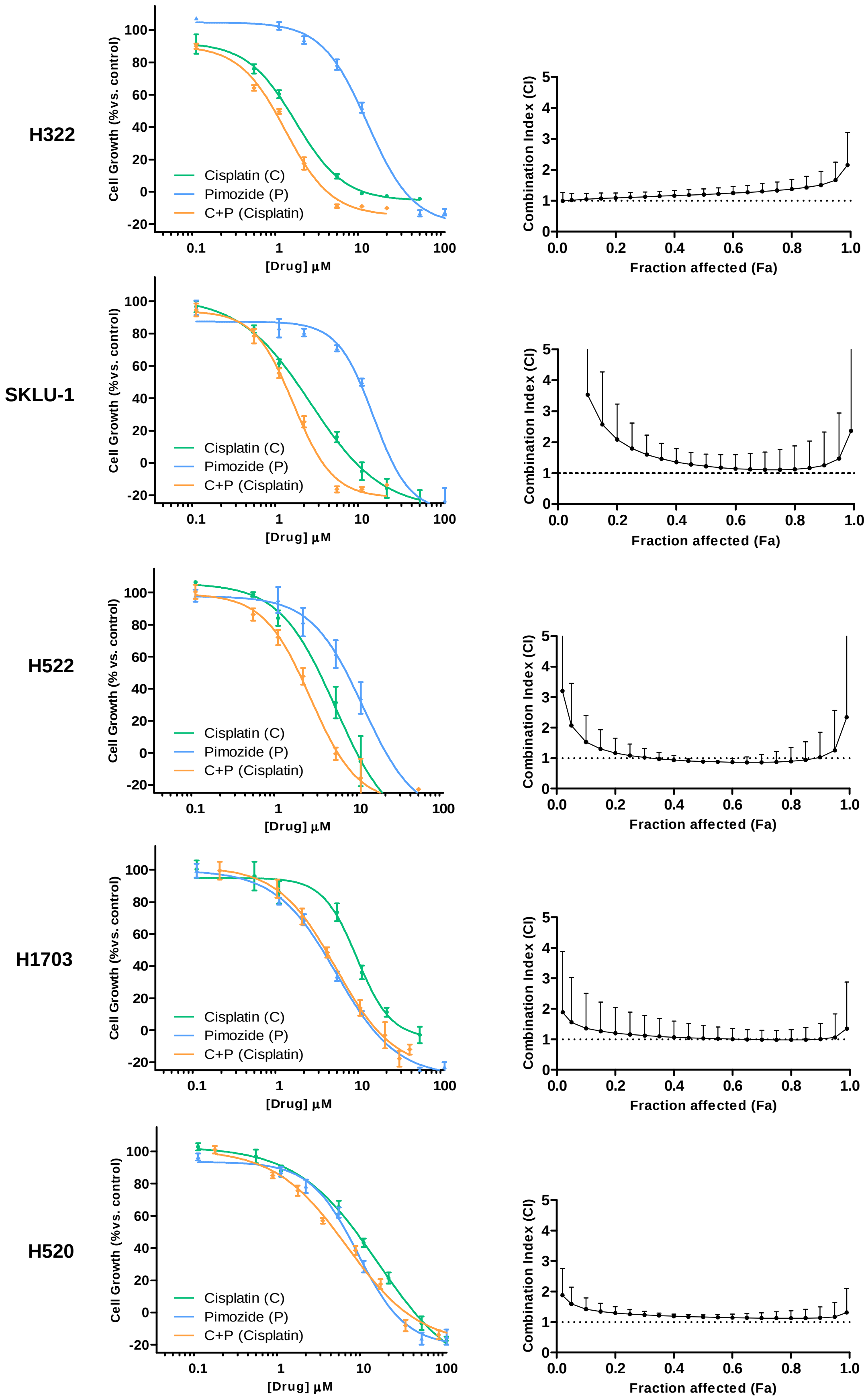

Supplementary Figure 1. Growth inhibition curves and the corresponding median-drug effect plots of five NSCLC cell lines treated with cisplatin, pimozide or their simultaneous combination. Exponentially growing NSCLC cells were treated and processed as described in Figure 4. Growth inhibition curves (left panels) were generated, where each point represents mean ± SEM of at least 3 replicates. IC50 values, the drug concentration that inhibits the cell growth by 50% (summarized in Table 3), were calculated by fitting the data to a sigmoid dose-response curve using GraphPad Prism. The treatment with the two drugs in combination was carried out using a constant ratio cisplatin:pimozide (as indicated in Table 3), which was established for each cell line as the ratio of the IC50 values for each single drug. The drug concentrations on the X-axis of the growth inhibition curves for the combination treatment refer to cisplatin. In order to evaluate the interaction between cisplatin and pimozide we used the median-drug effect analysis method originally described by Chou and Talalay. Using CalcuSyn software, the effect of the combination was compared to the effect of each drug to determine the combination index (C.I.) at a range of Fa (fraction affected by the dose) values. The resulting C.I. values were plotted against the Fa to produce the median-drug effect plot for each cell line (right panels).
